# Omeprazole Inhibits Proliferation and Modulates Autophagy in Pancreatic Cancer Cells

**DOI:** 10.1371/journal.pone.0020143

**Published:** 2011-05-24

**Authors:** Andrej Udelnow, Andreas Kreyes, Stefan Ellinger, Katharina Landfester, Paul Walther, Thomas Klapperstueck, Johannes Wohlrab, Doris Henne-Bruns, Uwe Knippschild, Peter Würl

**Affiliations:** 1 Department of General, Visceral and Transplantation Surgery, University Hospital of Ulm, Ulm, Germany; 2 Institute of Organic Chemistry, Macromolecular Chemistry and Organic Materials, University of Ulm, Ulm, Germany; 3 Max Planck Institute for Polymer Research, Mainz, Germany; 4 Department of Electron Microscopy, University of Ulm, Ulm, Germany; 5 Department of Dermatology and Venereology, Martin Luther University of Halle-Wittenberg, Halle, Germany; Florida International University, United States of America

## Abstract

**Background:**

Omeprazole has recently been described as a modulator of tumour chemoresistance, although its underlying molecular mechanisms remain controversial. Since pancreatic tumours are highly chemoresistant, a logical step would be to investigate the pharmacodynamic, morphological and biochemical effects of omeprazole on pancreatic cancer cell lines.

**Methodology/Principal Findings:**

Dose-effect curves of omeprazole, pantoprazole, gemcitabine, 5-fluorouracil and the combinations of omeprazole and 5-fluorouracil or gemcitabine were generated for the pancreatic cancer cell lines MiaPaCa-2, ASPC-1, Colo357, PancTu-1, Panc1 and Panc89. They revealed that omeprazole inhibited proliferation at probably non-toxic concentrations and reversed the hormesis phenomena of 5-fluorouracil. Electron microscopy showed that omeprazole led to accumulation of phagophores and early autophagosomes in ASPC-1 and MiaPaCa-2 cells. Signal changes indicating inhibited proliferation and programmed cell death were found by proton NMR spectroscopy of both cell lines when treated with omeprazole which was identified intracellularly. Omeprazole modulates the lysosomal transport pathway as shown by Western blot analysis of the expression of LAMP-1, Cathepsin-D and β-COP in lysosome- and Golgi complex containing cell fractions. Acridine orange staining revealed that the pump function of the vATPase was not specifically inhibited by omeprazole. Gene expression of the autophagy-related LC3 gene as well as of Bad, Mdr-1, Atg12 and the vATPase was analysed after treatment of cells with 5-fluorouracil and omeprazole and confirmed the above mentioned results.

**Conclusions:**

We hypothesise that omeprazole interacts with the regulatory functions of the vATPase without inhibiting its pump function. A modulation of the lysosomal transport pathway and autophagy is caused in pancreatic cancer cells leading to programmed cell death. This may circumvent common resistance mechanisms of pancreatic cancer. Since omeprazole use has already been established in clinical practice these results could lead to new clinical applications.

## Introduction

Despite relevant progression in diagnosis, resection and chemotherapy, pancreatic cancer is associated with a short survival [1]. Constitutive proliferation and profound resistance to apoptosis are characteristic features of pancreatic tumour cells rendering them highly resistant to common chemotherapeutic strategies. Several mechanisms responsible for apoptosis resistance have been reported including downregulation of proapoptotic proteins, upregulation of antiapoptotic proteins [2], [3], activation of various kinases such as protein kinase C (PKC)/ protein kinase D1 (PKD1) and casein kinase 1 (CK1) [4]–[6], elevated expression of various microRNAs [7], p53 mutations and mdm2 polymorphisms [8]. Therefore identification of substances which are able to circumvent these mechanisms would be valuable.

Recently, omeprazole (OMP), established as a world-wide standard drug for gastritis and duodenal ulcer since the 1980s, has been described as a potential antiproliferative agent and a resistance modulator both in vitro and in xenograft tumours of mice [9], [10]. Further research has suggested that inhibition of the vacuolar proton pump (vATPase), which regulates the lysosomal pH, or accumulation within the lysosomes may be the leading mechanisms for sensitising cells towards cytostatic treatment [9]–[12]. In addition, formation of reactive oxygen species [9] and involvement of p38 MAPK [13] have been reported to be associated with OMP-induced cellular effects. Furthermore P-glycoprotein (Pgp) [14] and cytochrome P450 2C19 isoform [15] cause pharmacokinetic interactions of OMP with other drugs (i.e. antibiotics, barbiturates, cytostatics) which are of clinical relevance. These data so far point to complex mechanisms involving, among others, the lysosomal transport system. The debate on whether and how OMP may inhibit cancer cell growth and enhance cytostatic effects of cytostatics are ongoing.

To date, neither the drug itself nor any of its targets have been directly observed within cancer cells. There is also virtually no data describing the dose-effect relationship of OMP in tumor cells. It would be of considerable interest whether this drug is effective in clinically applicable concentrations. Furthermore, to our knowledge, OMP has not yet been used in pancreatic cancer treatment, even though data obtained in patients with Zollinger-Ellison-syndrome show that OMP has a wide therapeutic range and causes only rare and mild side effects even at higher doses [16]. In contrast, other resistance modulators such as verapamil [17] or bafilomycin [18] are too toxic for clinical use.

Considering the high chemoresistance of pancreatic tumour cells, one of the main aims of our study was to determine whether OMP would be effective in pancreatic cancer cell lines. Therefore we investigated the one- and two-dimensional dose-effect relationships of OMP alone or in combination with 5-fluorouracile (5-FU) or gemcitabine (GEM) in the well characterized human pancreatic cancer cell lines MiaPaCa-2, ASPC-1, Colo357, Panc1, Panc89 and PancTu1 in vitro [19]–[21]. Our results indicate that the mean inhibitory concentrations (IC_50_) of OMP were in the range of clinical applicability in these cell lines.

For the further investigations we used the two cell lines MiaPaCa-2 and ASPC-1, and, beside of OMP, the cytostatic 5-FU in order to evaluate the specificity of the effects OMP causes within these cell lines.

We investigated the subcellular and molecular changes in MiaPaCa-2 and ASPC-1 cells treated with OMP. Transmission electron microscopy (TEM) and proton nuclear magnetic resonance spectroscopy of viable cells (H-NMRS) were performed. We found that modulation of autophagy is an early effect of OMP. Moreover, analysis of subcellular fractions containing lysosomes and Golgi complexes by Western blot analysis and NMR spectroscopy in untreated and treated MiaPaCa-2 cells confirmed that lysosomes are the main intracellular target of OMP leading to effects on protein recycling and transport pathways, including the Golgi complex.

Our results show that OMP inhibited the growth of pancreatic cancer cells in a dose-dependent manner and probably at non-toxic concentrations in vitro. OMP also enhances the effects of 5-FU and GEM. The lysosomal transport pathway is altered upon OMP treatment and autophagy is, directly or indirectly, modulated. Thus, OMP may provide a novel therapeutic approach in the treatment of pancreatic cancer.

## Results

### OMP inhibits pancreatic cancer cell proliferation in a dose dependent manner and enhances the cytostatic effects of GEM and 5-FU

The dose-effect curves of OMP, pantoprazole (PZL), GEM and 5-FU were generated using the ATP-bioluminescence assay. One of the aims of our investigations was to evaluate the clinical applicability of OMP. Therefore we determined the IC_50_ies by fitting the curves to a three parameter log-logistic model. The IC_50_, listed in table 1, ranged from 9–42 µg/ml for OMP, 22–99 µg/ml for PZL, 0.004–0.24 µg/ml for GEM and 0.20–0.57 µg/ml for 5-FU depending on the cell line.

**Table 1:** Mean inhibitory concentrations (IC_50_) of OMP and 5-FU in various human pancreatic cancer cell lines.

cell line	OMP		PZL		5-FU		Gemcitabine	
	IC	conf. int.	IC	conf. int.	IC	conf. int.	IC	conf. int.
MiaPaCa-2	42.4	38–45	68.4	57.8–79.7	0.20	0.12–0.27	0.021	0.013–0.028
ASPC-1	11.2	7.1–15.4	38.2	12.3–64.1	0.45	0.35–0.55	0.036	0.007–0.07
Panc-1	31.8	13.4–49.0	22.2	10.7–33.8	0.57	0.38–0.76	0.24	n.n.-0.57
Colo357	26.4	20.0–32.8	69.7	46.6–92.8	0.48	0.36–0.60	0.0032	0.0008–0.007
PancTu-1	20.7	5.9–35.4	43.8	25.1–62.5	0.17	0.08–0.26	0.035	n.n.-0.09
Panc-89	9.1	8.4–9.7	98.8	89.2–108.4	0.20	0.08–0.26	0.005	0.0005–0.01

The IC_50_ were determined by ATP-bioluminescence. All values are listed in µg/ml.

Abbreviations: conf. int. - 90% confidence intervals.

**Figure 1:**
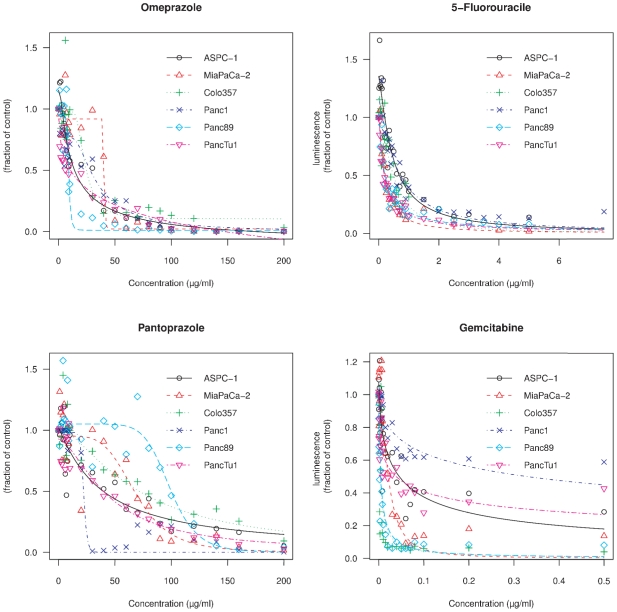
Dose effect curves of omeprazole, 5-fluorouracil, pantoprazole and gemcitabine in various pancreatic cancer cell lines. ASPC-1, MiaPaCa-2, Colo-357, Panc-1, Panc-89 and PancTu-1 cells were grown in the absence or presence of the indicated concentrations of omeprazole, 5-fluorouracile, pantoprazole or gemcitabine, respectively, for 4 days to determine the IC_50_ values. The IC_50_ ies and the sigmoidicities are gradually differing between the cell lines. In lower concentrations a slight growth-stimulatory effect (hormesis) was observed.

These dose-effect-curves (Figure 1) showed gradual differences of sigmoidicity and other pharmacodynamic parameters. It is obvious that in lower concentrations of the drugs the cell count may be even higher than in the respective control group (values above 1 in Figure 1) indicating a growth stimulatory effect. This phenomenon, called hormesis, which is defined as an overcompensatory reponse of living organisms to various stress factors [22], may account for resistance development during chemotherapy [23].

Therefore we investigated the effects of OMP when combined with a cytostatic in lower concentrations in order to evaluate its role in chemoresistance and hormesis overcome. Additive, synergistic or antagonistic mutual interactions of two drugs can be quantified within the quasilinear region of the dose-effect-curves using the median effect principle of Chou [24] or the unified response surface area of Greco et al. [25]. However the interactions of drug combination is difficult to quantify for hormetic dose-response-relations [26]. There are various models to assess hormesis for a single drug treatment [27], [28].

**Figure 2:**
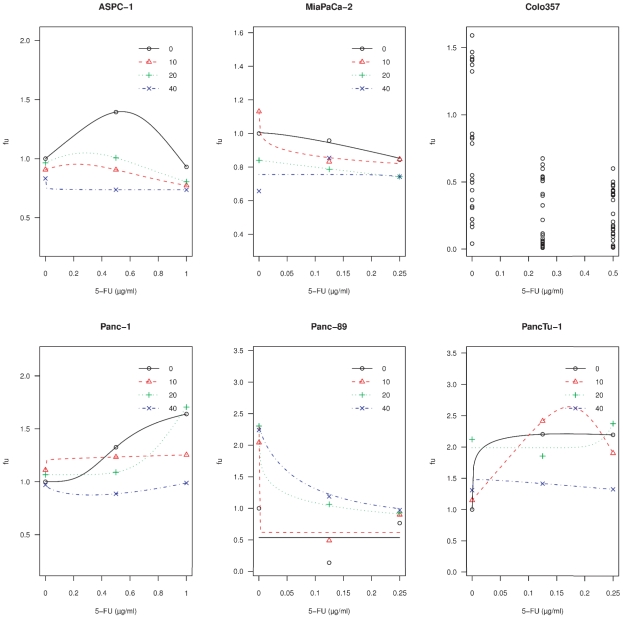
Hormesis of low-dose 5-FU in pancreatic cancer cells and interaction with OMP. The cell lines ASPC-1, MiaPaCa-2, Colo357, Panc-1, Panc89 and PancTu1 were untreated or treated with 5-FU alone or in combination with the indicated concentrations of OMP for 4 days. At lower doses of 5-FU alone (black lines, 0 µg/ml OMP) a growth-stimulatory effect (hormesis) was observed in the cell lines ASPC-1, Panc-1 and PancTu-1. The data points show the means of 8 measurements. The curves are fitted in these cell lines using the Brain-Cousens model (see sub-section 4.11). In MiaPaCa-2 and Panc-89 cells no hormesis occured, these curves were fitted using a three parameter logistic model. In Colo357 cells the curves could not be fitted by any pharmacodynamic model due to large standard errors at these lower concentrations (data points shown). The red, green and blue lines indicate the dose-effect curves of 5-FU when various concentrations of OMP were added (10, 20 and 40 µg/ml, respectively). In ASPC-1 and Panc-1 cells the hormesis of 5-FU was reversed and in PancTu-1 cell it was mitigated by OMP depending on the concentration of 5-FU. In MiaPaCa-2 cells we found an additive interaction of 5-FU with OMP. In Panc-89 cells the interaction was antagonistic.

Table 2 shows the unaffected fraction (f_u_), which is the cell count related to control, upon low concentrations of 5-FU and GEM when used as single agents. In ASPC-1, Panc-1 and PancTu-1 cells significant elevations of the f_u_ above 1 upon 5-FU, indicating hormesis, were observed. In contrast there was no significant hormesis upon GEM. When OMP is added to 5-FU in these concentrations, the hormesis is mitigated in the ASPC-1, Panc-1 and PancTu-1 cell lines (Figure 2). In MiaPaCa-2 cells the OMP and 5-FU combination showed additive effects, in the Panc-89 cell line OMP led to an antagonistic interaction. In Colo357 cells no dose-effect-curve could be established due to large standard errors. GEM and OMP showed additive or antagonistic interactions (data not shown).

**Table 2:** Detection of growth-stimulatory effects of 5-FU and GEM in lower doses.

Cell line	5-FU			GEM		
	c (µg/ml)	f_u_	p (t-test)	c (µg/ml)	f_u_	p (t-test)
ASPC-1	0.5	1.4	0.047	0.02	0.5	0.002
	1	0.9	0.38	0.04	0.4	0.007
MiaPaCa-2	0.125	0.9	0.4	0.01	0.36	0.01
	0.25	0.8	0.2	0.02	0.15	0.003
Colo357	0.5	0.3	0.02	0.02	0.9	0.2
	1	0.2	0.01	0.04	0.8	0.06
Panc-1	0.5	1.3	0.02	0.01	0.5	0.0001
	1	1.6	0.0003	0.02	0.4	<0.0001
Panc-89	0.125	0.2	0.01	0.002	0.7	0.02
	0.25	0.9	0.3	0.004	0.6	0.04
PancTu-1	0.125	2.2	0.005	0.02	0.35	0.01
	0.25	2.2	0.0001	0.04	0.3	0.01

Significant elevations of cell counts compared to the control groups could be observed upon 5-FU in ASPC-1 (at 0.5 µg/ml 5-FU), Panc-1 (at 0.5 and 1 µg/ml 5-FU) and PancTu-1 (at 0.125 and 0.25 µg/ml) cells. In contrast, no hormesis could be observed upon GEM.

Abbreviations: c-concentration, fu-unaffected fraction (cell count related to control).

Thus, OMP has a dose dependent antiproliferative effect and the in vitro IC_50_ of OMP in these support the hypothesis of clinical applicability (which is discussed further in the discussion section). However, the dose-effect relationship of the drugs differed between cell lines. The combined treatment of OMP with either 5-FU or GEM revealed dose-dependent additive interactions and a mitigation of the hormetic growth stimulation of low-dose 5-FU in ASPC-1, Panc1 and Panc89 cells. .

### Cell line specific changes in the intralysosomal pH after treatment of pancreatic cancer cells with OMP and 5-FU either alone or in combination

Acridine orange (AO) fluorescence microscopy was performed to determine whether OMP increases the intralysosomal pH as described in recent reports, either by inhibiting the vATPase [10] or by accumulating within the lysosomes [11]. Acidic cell compartments are stained red and neutral compartments are stained green by AO. Specific inhibition of the vATPase by bafilomycin A1 has been shown to lead to a rapid inhibition of the red fluorescence [29], [30].

**Figure 3:**
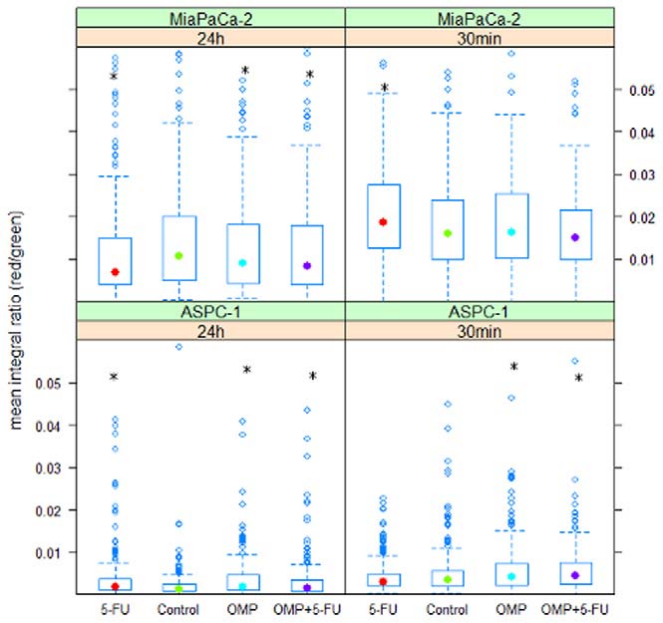
Box-Whisker plots of lysosomal acidity quantified by AO fluorescence microscopy and image analysis. Acridine Orange was added to living untreated ASPC-1 and MiaPaCa-2 cells and cells treated with 5-FU, OMP or the combination of both for 30 minutes or 24 hours. Microscopical life images were taken at 525 nm (green) and 650 nm (red) to detect changes in the lysosomal pH value (three images per plate, three plates per group). The red to green fluorescence ratio of the lysosomes of treated cells were compared to the control groups by the Mann-Whitney-U-test. Significant differences compared to control are marked by *. In ASPC-1 cells, after 30 min of treatment, intralysosomal acidity increased upon treatment with OMP (p:0.0051) and 5-FU+OMP (p<0.0001). After 24 hours, the acidity is increased upon all treatment regimens (5-FU - p:0.0002; OMP - p<0.00001; OMP+5-FU - p:0.037). In MiaPaCa-2 cells the acidity is elevated after 30 min upon 5-FU (p:0.005) and decreased after treatment with OMP (p:0.037), 5-FU (p:0.00026) and 5-FU+OMP (p:0.011) after 24 hours.

The red-to-green fluorescence ratios quantified by quantitative image analysis for MiaPaCa-2 and ASPC-1 cells are shown in Figure 3. The early changes (after 30 minutes of treatment) consisted of a slightly higher acidity upon 5-FU treatment in MiaPaCa-2 cells and in ASPC-1 cells treated with OMP or OMP+5-FU. After 24 hours, the lysosomal acidity of MiaPaCa-2 cells was gradually, but significantly suppressed by OMP, 5-FU and OMP+5-FU. However these observation do not unequivocally correspond to the above mentioned reports, since the effect was not specific to OMP. In ASPC-1 cells, all three treatment regimens resulted in a slightly higher acidity after 24 hours.

Thus we conclude that OMP did not cause a consistent change in the intralysosomal pH value of pancreatic cancer cells. Nevertheless a slight inhibition occured in MiaPaCa-2 cells upon all treatment regimens after 24 hours. In contrast, ASPC-1 cells showed a higher lysosomal acidity when treated with OMP, 5-FU or a combination of both.

### Phagophores and autophagosomes accumulate in ASPC-1 and MiaPaCa-2 cells after OMP treatment

So far, our results suggest that vATPase inhibition and lysosomal pH elevation were not the main effects caused by OMP in MiaPaCa-2 and ASPC-1 cell lines. The AO fluorescence microscopy however revealed that lysosomes were involved in both OMP- and 5-FU-mediated inhibition of proliferation. Therefore we examined the subcellular morphological changes after treatment with OMP, 5-FU and the combination of both by TEM.

**Figure 4:**
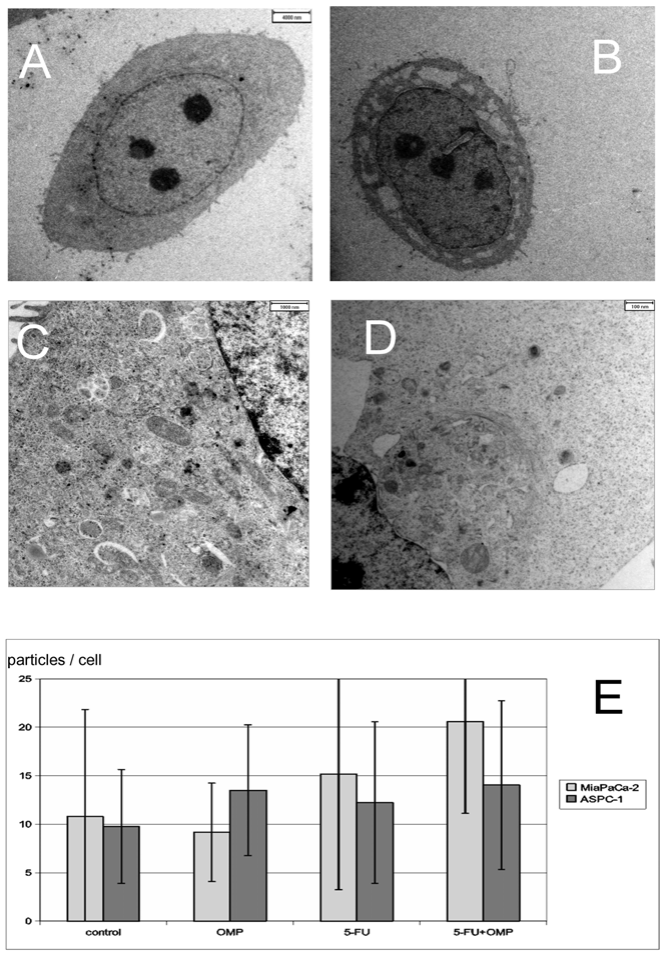
Electron microscopy of the ASPC-1 and MiaPaCa-2 cell lines treated or untreated with OMP. (A) ASPC-1 cell without treatment (800 fold). (B) ASPC-1 cell undergoing apoptosis upon 160 µg/ml OMP after 24 hours (800 fold). Vacuolisation of the cytoplasma and condensation of the nucleus are visible. (C) Phagophores and autophagosomes in a segment of an ASPC-1 cell treated with omeprazole 80 µg/ml for 24 hours (2800fold enlargement). The phagophores are characterised by a cup-like shape (white arrows). Autophagosomes are closed particles, the number of which is increased in treated cells (black arrows). (D) Early phagophores and autophagosomes are also found in MiaPaCa-2 cells treated with OMP 80 µg/ml after 24 hours in a perinuclear region containing lysosomes and the Golgi complex. In contrast to ASPC-1 cells, early signs of apoptosis such as vacuolization, are also present. (E) Barchart of the numbers of autophagosomes and lysosomes per cell in MiaPaCa-2 and ASPC-1 cells untreated or treated with 5-FU, OMP or the combination of both for 24 hours with standard errors. Significant differences compared to control are marked by *. In ASPC-1 cells there were significant differences compared to the control in the OMP group (p: 0.03) and the 5-FU+OMP group (p: 0.03). In MiaPaCa-2 cells the 5-FU+OMP group differed significantly from control (p<0.001).

Figure 4 compares an untreated ASPC-1 cell (Figure 4 A) with cells treated with 160 µg/ml (Figure 4B) and with 80 µg/ml OMP (Figure 4C) for 24 hours. Treatment with 80 µg/ml OMP resulted in autophagy as indicated by the appearance of early cup-like phagophores containing cytoplasmic material and autophagosomes filled with coated vesicles (Figure 4C). After treatment with 160 µg/ml OMP, the ASPC-1 cells underwent apoptosis after 24–48 hours (figure 4B). Similar changes were observed in MiaPaCa-2 cells treated with 80 µg/ml OMP including vacuolisation as a concomitant sign of apoptosis (Figure 4D).

Although phagophores were easily identified by unique morphological characteristics, autophagosomes, lysosomes, endosomes and autolysosomes could not be distinguished by TEM. Significantly higher amounts of all these lysosome-like organells were observed in ASPC-1 cells treated with OMP or OMP+5-FU and in MiaPaCa-2 cells treated with OMP+5-FU (Figure 4E).

In summary, OMP led to accumulation of markers of early stages of autophagy (autophagophores) in both cell lines. Although markers of later stages, such as autolysosomes, could not be distinguished morphologically from other lysosomal transport pathways, the overall number of lysosome-like organelles increased in ASPC-1 cells upon treatment with OMP or 5-FU+OMP and in MiaPaCa-2 cells treated with 5-FU+OMP. These findings suggest that either activation of the protein turnover or impairment of the lysosomal transport pathway occurred.

### Identification of metabolic changes within viable cells after treatment with OMP, 5-FU or a combination of both

**Figure 5:**
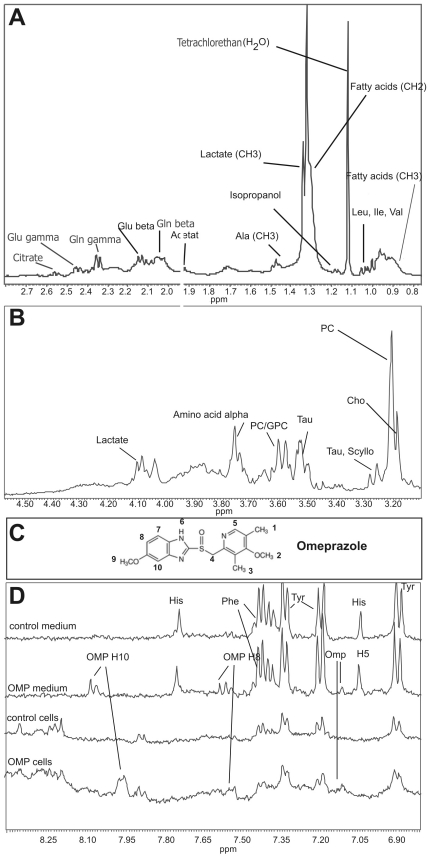
Identification of substances from a proton NMR spectrum of viable untreated MiaPaCa-2 cells. The cells were harvested from monolayer culture, kept and measured at 20°C. Measurement were performed by a 600 MHz Bruker spectrometer. For better visibility the part of the spectrum showing the protons of aliphatic groups is splitted into 2 parts - A and B. (A). aliphatic part I. The methyl and β- and γ- methylene groups of various fatty acids and amino acids are visible. In addition, isopropanol and tetrachlorethan (the external concentration standard) occured as pollutions. (B) Aliphatic part II. Phospholipid metabolites and the α-methylene groups of amino acids and lactate are visible. (C) Formula of OMP with numbering of the respective protons. The methyl groups (1–3, 9) and the methyl group (4) are covered by other metabolites in the aliphatic parts of the spectrum. In contrast, the aromatic protons are visible (H5, H8, H10). (D) Overlay of the aromatic parts of different spectra for intracellular identification of OMP. The singulet of the H5 proton and the dublets of the H8 and H10 protons can be identified when the medium and the cell spectra are compared to those without OMP treatment. Abbreviations: His - histidine, Tyr - tyrosine, Phe – phenylalanine, Leu, Ile, Val - Leucine, Isoleucine, Valine. Ala - Alanine. Glu - Glutamate, Gln – Glutamine, PC - phosphatidylcholine, Cho - Choline, GPC – glycerophosphocholine, Tau – taurine, Scyllo - scylloinositole.

Proton NMR spectroscopy of viable cells was performed in MiaPaCa-2 and ASPC-1 cell lines in order to analyse the biochemical processes associated with the morphological changes described above. Various characteristic low-molecular intracellular metabolites such as fatty acids, amino acids, membrane associated phospholipid mebolites and intermediate citrate cycle and glycolysis metabolites were identified (Figure 5).

OMP itself was detected intracellularly within both cell lines after treatment with 80 µg/ml for 24 hours. The signals of the aromatic H8 and H10 protons of OMP (Figure 5C) were easily identified (Figure 5D) in MiaPaCa-2 cells. OMP was also identified within ASPC-1 cells by the H5 proton (data not shown). To our knowledge, this is the first time that OMP has been observed within cells.

**Figure 6:**
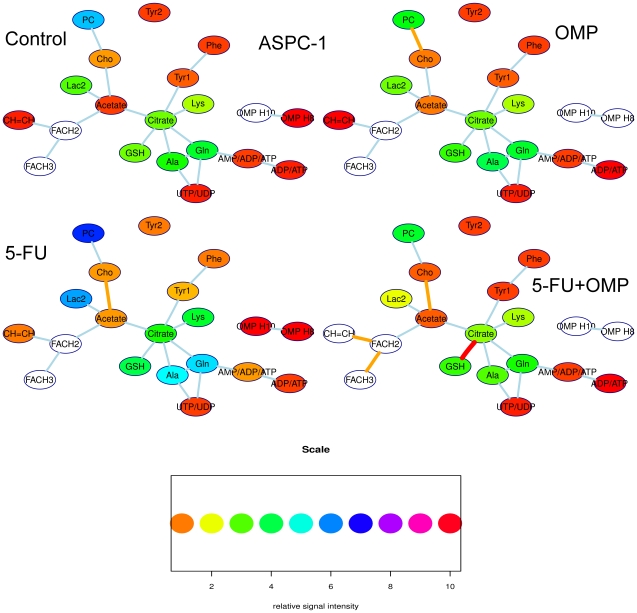
Simplified metabolic networks of ASPC-1 cells as determined by proton NMR spectroscopy. The ASPC-1 cell line is shown without and upon various treatment regimens (OMP, 5-FU or 5-FU+OMP combination). The nodes of this network symbolize metabolite signals, their colours correspond to relative signal intensity (when compared to an external standard) as indicated in the heatmap scale below. The signal intensity is linearly related to the intracellular concentration. The background of the nodes are left blank when the signal intensity is out of the range indicated by the heatmap scale. The lines between the boxes symbolize strongly simplified metabolic pathways. The colours of these lines indicate significant differences of the signal intensity ratios of the connected metabolites compared to the control group when orange (p<0.05), red lines indicate p<0.01. The most obvious changes is that the PC/Cho ratio is significantly lower in the OMP group compared to the control group. Upon 5-FU, the Cho/Acetate ratio decrease is the only significant change. In the 5-FU+OMP group, there are several significant changes, i.e.the FACH2/CH = CH ratio is significantly higher. Moreover, the Cho/Acetate ratio changed upon 5-FU+OMP as in the 5-FU group, but also the citrate/GSH ratio. The cellular biochemical effects involve mainly the fatty acid and phospholipid metabolism pointing to membrane anabolism. Abbreviations: Gln - glutamine, Ala - alanine, PC - phosphatidylcholine, Cho – Choline, Lac1+FACH2 - methyl group signal of lactate and methylene groups of the fatty acids, Lac2 - methylene group of lactate, CH = CH - protons of methin groups of unsaturated fatty acids.

**Figure 7:**
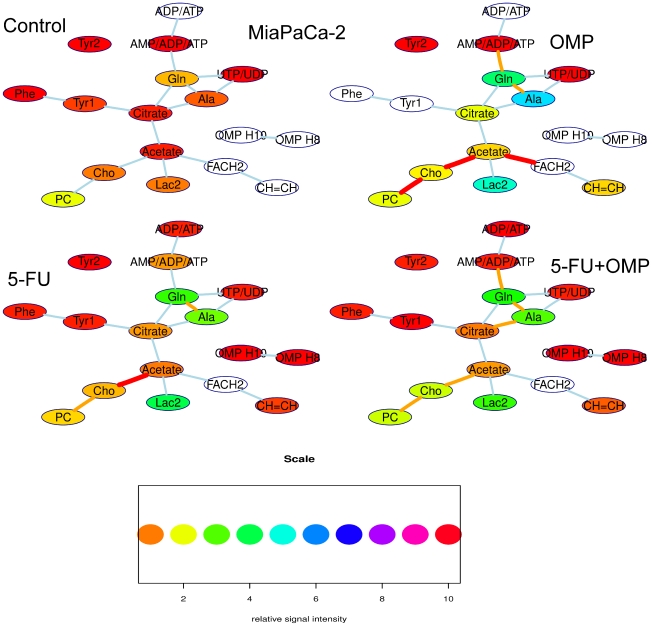
Simplified metabolic network of the MiaPaCa-2 cell line as determined by proton NMR spectroscopy. The cell line is shown without and upon various treatment regimens (OMP, 5-FU or 5-FU+OMP combination). The nodes of this network symbolize metabolite signals, their colours correspond to relative signal intensity (when compared to an external standard) as indicated in the heatmap scale below. The signal intensity is linearly related to the intracellular concentration. The background of the nodes are left blank when the signal intensity is out of the range indicated by the heatmap scale. The lines between the boxes symbolize strongly simplified metabolic pathways. The colors of these lines indicate significant differences of the signal intensity ratios of the connected metabolites compared to the control group when orange (p<0.05), red lines indicate p<0.01. Upon OMP, the PC/Cho ratios are significantly lower compared to control. Furthermore the acetate/FACH2 ratio is significantly decreased in the OMP group. The latter also showed a higher CH = CH level, the ratio to FACH2 is, however, decreased. Upon 5-FU and 5-FU+OMP, similar changes could be observed. Furthermore, in contrast to ASPC-1 cells, the Ala/Gln/AMP pathway is also involved. Abbreviations: Gln - glutamine, Ala - alanine, PC - phosphatidylcholine, Cho - Choline. Lac1+FACH2 - methyl group signal of lactate and methylene groups of the fatty acids, Lac2 - methylene group of lactate, CH = CH - protons of methin groups of unsaturated fatty acids.

Although the determination of absolute intracellular concentrations by NMR spectroscopy is generally prone to systematic errors, the calculation of integral ratios of various signals may contain useful quantitative information on the metabolic pathways. These metabolic pathways are shown as a strongly simplified network for ASPC-1 cells in Figure 6 and MiaPaCa-2 cells in Figure 7. A line between two signals symbolises a metabolic pathway. The line is orange when the signal intensity ratio of the connected substances differs from the control with p<0.05 and red when p is below 0.01.

Membrane-bound fatty acids are usually NMR-visible only when using the Magic-Angle-Spinning (MAS) technique [31]. In contrast, mobile polyunsaturated fatty acid (PUFA) groups (viz. the C**H** = C**H** at 5.3 ppm or CH = CHC**H2**CH = CH at 2.8 ppm), which have been associated with the formation of lipid drops in autophagosomes during autophagy and programmed cell death (PCD) [32], became prominent in the proton NMR spectra of MiaPaCa-2 cells treated with OMP or 5-FU+OMP. Moreover an increase in the fatty acid methylene (FACH2) groups compared to acetate was observed upon OMP and 5-FU+OMP treatment in MiaPaCa-2-, but not in ASPC-1 cells. This correlates with the electron microscopy data showing that the morphological changes associated with autophagy are accompanied by vacuolisation which points to the beginning of apoptosis in MiaPaCa-2, but not ASPC-1 cells, at 80 µg/ml OMP.

Cho and Phosphocholine (PC) signals are indicators of growth in human cells, whereby elevated PC is associated with rapid proliferation and malignant behaviour [33]–[35]. The suppression of PC may be caused by choline kinase and phospholipase C downregulation and phospholipase A2 induction leading to proliferation inhibition [36]. In our study, PC was significantly suppressed compared to Cho in MiaPaCa-2 cells treated with OMP, 5-FU and 5-FU+OMP and in ASPC-1 cells treated with OMP alone.

Taken together, these data show that the fatty acid and phospholipid metabolite signals changed upon OMP treatment. Although similar changes were observed in both ASPC-1 and MiaPaCa-2 cells, these changes were more enhanced in MiaPaCa-2 cells, especially with respect to those signal changes indicating inhibited proliferation (PC) and PCD (PUFA). This corresponds to the above described pharmacodynamic and morphologic changes. However in MiaPaCa-2 cells the biochemical effects were not limited to OMP treatment, but also occured upon 5-FU and 5-FU+OMP and thus can not be considered to be specific to OMP.

### Involvement of lysosomes and the Golgi complex in the cellular response to OMP

**Figure 8:**
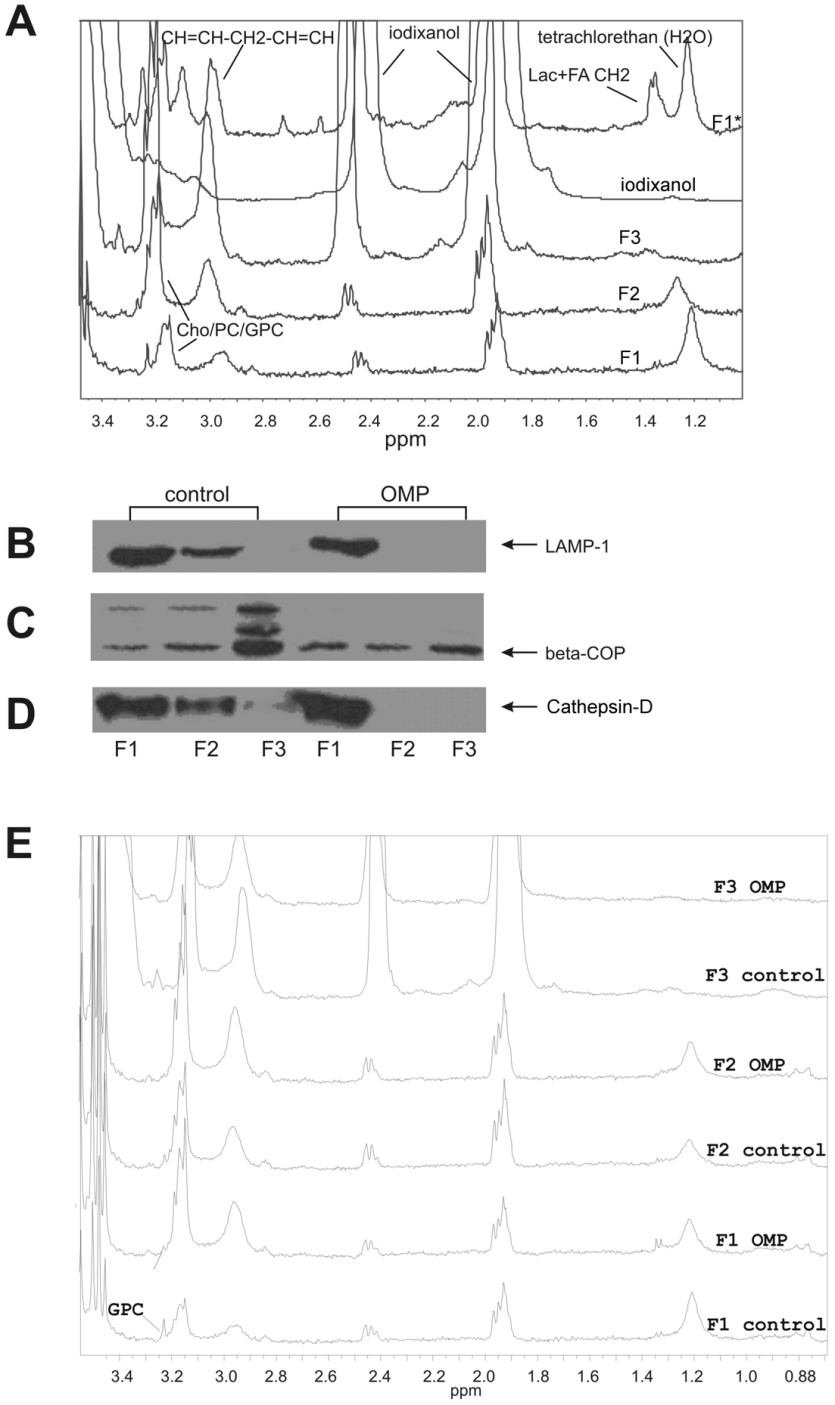
Analysis of MiaPaCa-2 cell organelles after ultracentrifugation. (A) Overlay of the proton NMR spectrum fragments of the following suspensions (from top to down): untreated first (lysosomal) cell fraction after ultracentrifgation and one washing (F1*), iodixanole (Optiprep) suspension, lower (F3), middle (F2) and upper fraction (F1) of the ultracentrifugates after two washings. The chemical shifts of the spectra were calibrated to the Choline/Phosphatidylcholine (Cho/PC) signal. Apart from iodixanol, we could identify the PUFA groups (methylene groups situated between two CH = CH groups), Cho/PC and glycerophosphocholine (GPC) in F1* and F1–3. Furthermore we observed lactate and the methylene groups of fatty acids (at 1.3 ppm), citrate (at 2.55 and 2.7 ppm) and phosphoethanolamine (at 3.1 ppm) in the F1* but not in the F1–F3 groups.. (B) Western blot analysis of LAMP-1, a late endosome marker, which was nearly identically distributed over the first two fractions compared to Cathepsin-D, in the controle group, but ocurred only in the first fraction in the OMP treated group. β-COP as an indicator of the Golgi-complex, is found strongly in the lower fraction in the control group, but very weakly in the OMP treated group. Cathepsin - D, an early endosome marker, which can be found in the upper and middle fraction of the control group, but regarding the OMP group, it was only found in the first one. (E) Overlay of NMR spectra of all fractions of control and OMP. The most relevant difference is the weaning of the GPC signal after OMP treatment in the 1st fraction after only 6 hours.

Potential subcellular targets of OMP were identified by isolating organelles from MiaPaCa-2 cells by density centrifugation. As shown in Figure 8, the first and second density fractions contained early and late endosomes and lysosomes which were identified by LAMP-1 and Cathepsin-D antibodies (Figure 8B) in the control group. In the OMP treated group, these proteins were found only in the first fraction. The third fraction of the control group contained the Golgi complex as indicated by the β-COP antibody. However, expression of this protein diminished upon OMP treatment. The proton NMR spectra of these fractions are shown in figures 8A and 8C. Figure 8A shows the signal assignments comparing the spectra of these fractions (named F1–F3) to those of iodixanol (Optiprep), and a lysosomal cell fraction of untreated cells (F1*), which was washed with PBS once after ultracentrifugation (in contrast to fractions F1–F3, which were washed twice). Iodixanol occured in all fractions, but decreased after washing the ultracentrifugates twice. This points to partial incorporation of iodixanol into the membranes or the lumen of the organelles. Most of the water-soluble lower-molecular substances decreased also in F1–F3, such as lactate and citrate, compared to F1*, which pointed to a washout. The integrity of the organelles were veryfied by using an external standard (tetrachlorethan) for chemical shift calibration. There was a pH-dependent shift of the citrate signals towards acidification in the lysosomal fractions compared to the whole cell suspensions.

In contrast to the whole cell spectra (see figure 5B), glycerophosphocholine (GPC) was found in the first (lysosomal) fraction. When comparing the control to the OMP-treated group (Figure 8C), the GPC signal clearly diminished upon OMP treatment. Furthermore, an increase in PUFAs in the OMP-treated group was confirmed (Figure 8C) analogously to the whole cell spectra. OMP could not be identified in any of these fractions and therefore does probably not accumulate in lysosomes or in the Golgi complex. This underscores the finding that the endosome and lysosome fractions underwent anabolic changes during OMP treatment (corresponding to the TEM data). However, the Golgi complex as a substantial part of the lysosomal transport system, is also involved.

### Determination of autophagic activity

We performed LC3-Western blot analysis to detect changes in the expression of LC3. The LC3-gene expression correlates well with the number of autophagosomes [37], [38]. Furthermore two fractions can usually be differentiated - LC3-I and LC3-II. The first occures in autophagophores and is considered an autophagy induction indicator, the latter is contained within the autophagosomes and is degraded, after their fusion with lysosomes, in autolysosomes. Therefore, by using specific inhibitors of the autophagic pathway at the level of this fusion, such as Bafilomycin A1, the induction may be differentiated from the turnover [37], [38].

**Figure 9:**
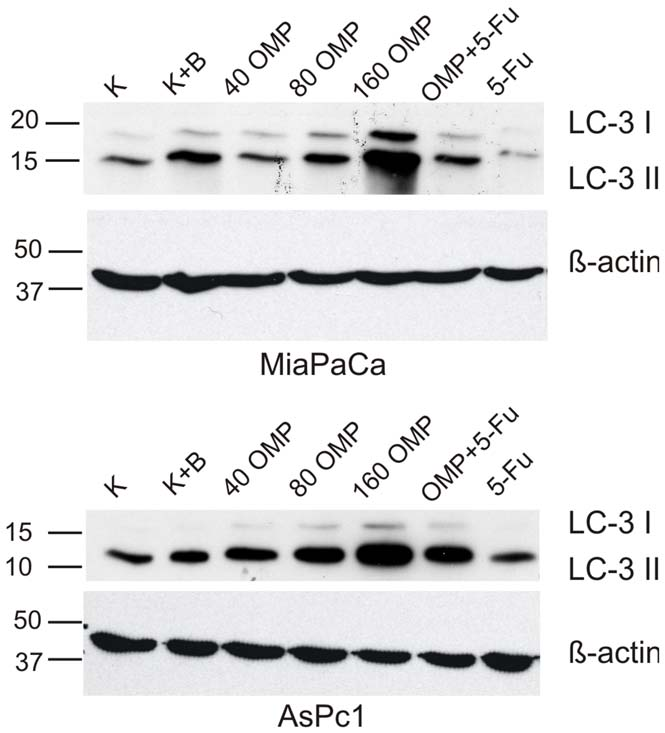
Western Blot analysis with LC3B-antibody in ASPC-1 and MiaPaCa-2 cells. The experiment was repeated three times with nearly identical results. The LC3-I fractions can be clearly distinguished above the LC3-II signals. Bafilomycin A1 elevated slightly the LC3-II signal intensity compared to control in both cell lines. There was a marked and dose-dependent increase of the signal strength of both fractions when OMP was used. 5-FU alone did not relevantly influence the LC3-level. The corresponding β-Actin level is shown below the LC3-WB to confirm the correctness of this semiquantitative evaluation.

Figure 9 shows the results of the Western blot analysis. Treatment with Bafilomycin A1 was performed for only 4 hours, since long-term treatment may lead to cytotoxicity which could compromise or mask the specific effects of Bafilomycin A1 [37]. In contrast, OMP and/or 5-FU was added to the medium for 24 hours. Bafilomycin A1 led to a slightly increased LC3-II signal, while the LC3-I-fraction was not changed. This corresponds to the already known mechanisms of action [37] and indicates an accumulation of autophagophores resulting from impaired turnover. In contrast, OMP led to a dose-dependent marked elevation of both the LC3-I and the LC3-II fractions in MiaPaCa-2 and ASPC-1 cells which points to both strong autopagy induction and decreased turnover. Although the LC3-II fraction is seemingly overproportionally increased compared to the LC3-I fraction, this ratio was not quantified because of the known artifacts the LC3-I fraction may undergo [38]. The possible role of autophagy in the apoptosis induction upon OMP is further addressed in the Discussion section. 5-FU did not relevantly influence the autophagic activity and the combination treatment reflects the effects of OMP. We conclude that OMP induced autophagy in a dose-dependent manner (indicated by an elevated LC3-II level) and in the mean time obviously led to an accumulation of the LC3-II level pointing to impaired autophagosome turnover at the fusion level. This corresponds to the findings described above.

### Comparison of further gene expression patterns in MiaPaCa-2 and ASPC-1 cells treated with OMP

**Figure 10:**
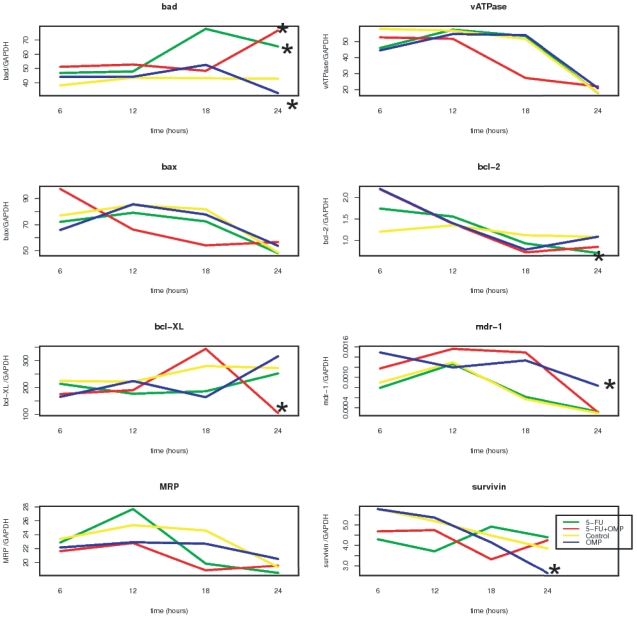
Gene expression analysis of membranal transport and apoptosis relevant genes in ASPC-1 cells. Quantification of mRNAs in ASPC-1 cells untreated or treated with OMP 80 µg/ml, 5-FU 5 µg/ml or the combination of both was performed at different time points throughout 24 hours, and the means of three replicates are shown for every time point. The stars indicate significant differences after 24 hours compared to control. The pro-apoptotic bad-mRNA was upregulated after 5-FU- and 5-FU+OMP-treatment. The antiapoptotic bcl-2 mRNA was downregulated by 5-FU and bcl-XL by 5-FU+OMP. OMP led to downregulation of bad and survivin and to upregulation of the mdr-1 mRNA.

Although the above described mechanisms may explain some of the early events that occur after OMP treatment, how they cause antiproliferative effects still remained unclear. Therefore, we compared the expression of pro- and antiapoptotic genes as well as membrane transport protein genes in untreated, OMP- and/or 5-FU treated cells. The mRNAs of mdr-1, vATPase, mrp, bcl-2, bcl-xL, bax, bad and survivin genes in ASPC-1 cells were quantified throughout the first 24 hours of treatment (Figure 10). The pro-apoptotic bad mRNA was up-regulated by 5-FU (p: 0.038) and 5-FU+OMP (p: 0.0033) and suppressed by OMP (p: 0.028) in ASPC-1 cells (after 24 hours). The anti-apoptotic bcl-2 mRNA was downregulated by 5-FU (p: 0.022), but not by treatment with OMP or 5-FU+OMP. Hence, we assumed that 5-FU induced apoptosis rather than autophagy. In contrast, the antiapoptotic bcl-xL mRNA was significantly downregulated by 5-FU+OMP (p:0.0017). The mdr-1 mRNA, which codes for the membrane Pgp and allows the cell to export various toxins, such as OMP, was significantly upregulated after treatment with OMP for 24 hours (p: 0.019). However, the mRNA expression of mrp, which is not known to interact with OMP, did not differ from the control. Survivin mRNA was significantly downregulated by OMP (p: 0.0059). Survivin promotes cancer progression and survival, and its suppression is one of the prerequisites of successful cancer treatment [39]. Cells treated with a combination of 5-FU (5 µg/ml) and OMP (80 µg/ml) elicited similar responses to cells treated with 5-FU alone rather than with OMP alone. Upregulation of mdr-1 and down-regulation of survivin were the most specific gene expression changes observed upon OMP treatment.

**Figure 11:**
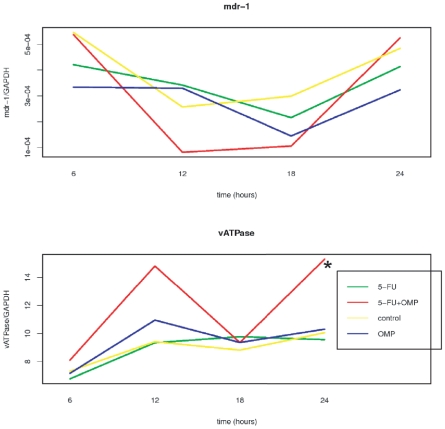
Gene expression analysis of membranal transport and apoptosis relevant genes in MiaPaCa-2 cells. mRNA quantification in MiaPaCa-2 cells untreated or treated with OMP 80 µg/ml, 5-FU 5 µg/ml or the combination of both was performed at different time points throughout 24 hours, and the means of three replicates are shown for every time point. While the mdr-1 mRNA is not signicantly changed, the vATPase mRNA is upregulated in the 5-FU+OMP group after 24 hours compared to control.

Mdr-1 and vATPase mRNAs were also quantified in MiaPaCa-2 cells (Figure 11). While the mdr-1 mRNA remained unchanged in the treatment groups compared to the control group after 24 hours, the vATPase mRNA was significantly upregulated by 5-FU+OMP.

**Figure 12:**
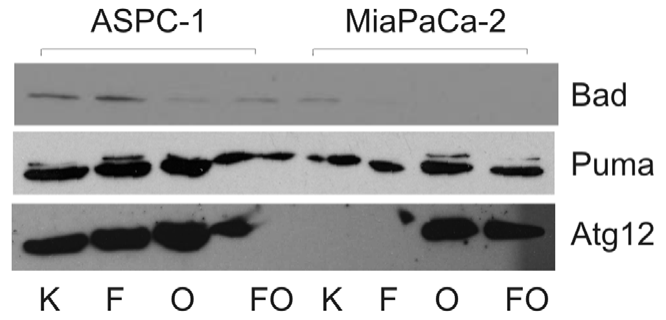
Gene expression analysis of autophagy and apoptosis relevant genes by Western blot analysis. The Bad, Puma and Atg12 proteins were detected in MiaPaCa-2 and ASPC-1 cells. The Bad expression is slightly increased by 5-FU and suppressed by OMP and 5-FU+OMP in ASPC-1, but not in MiaPaCa-2 cells. Puma is induced by OMP in both cell lines indicating involvement of the BH3-only pathway. Atg12 is strongly enhanced by OMP in both cell lines. Abbreviations: K - control, F - 5-FU, O - OMP, FO - 5-FU+OMP.

Western blot analysis revealed that atg12, another specific marker of autophagy [40], [41], was strongly activated upon OMP treatment in MiaPaCa-2 and ASPC-1 cells and upon 5-FU+OMP treatment in MiaPaCa-2 cells (Figure 12). Protein expression of puma, a marker of the BH3-only apoptotic pathway, was up-regulated in ASPC-1 cells by OMP, but not in MiaPaCa-2 cells (Figure 12).

## Discussion

The enhancement of chemotherapeutic effects as well as proliferation inhibition and apoptosis induction by OMP have been described as early as 1999 [10], [11], [42], [43]. To date only one clinical investigation has reported a potential antitumor effect in patients. Treatment of gastric MALT-lymphoma by a Helicobacter pylori eradication scheme containing 20 mg OMP once daily per os resulted in tumour regression [44]. However Katagiri et al [45] have demonstrated that oral doses of 20 mg OMP led to a maximal plasma concentration of about 2.5 µg/ml (7 µM) after two hours in patients. This gastroprotective dose would probably not be sufficient for an antitumor effect in pancreatic cancer since our in vitro studies demonstrated that concentrations greater than 30 µg/ml are required to elicit an effect.

Treatment of Zollinger-Ellison-Syndrome with up to 120 mg OMP three times a day has rare and mild side effects [16] and no long-term effects [46] (corresponding to a hypothetical in-vivo peak concentration of about 15 µg/ml). This is in the range of the IC_50_ of most cell lines studied here, but overdoses up to 2400 mg (corresponding to a hypothetical plasma peak concentration of 300 µg/ml) have been tolerated with minor side effects (AstraZeneca product information). Thus we hypothesise that if OMP was to elicit an antitumoral effect in patients, the necessary dose would possibly be in the tolerable range. While recently published in vivo data confirmed an antitumoral effect for human melanoma and colon adenocarcinoma cell lines in SCID mice [10], a phase I clinical investigation has not yet been performed.

OMP is a prodrug which upon activation blocks gastric parietal cell proton pumps by disulfid formation in an acid environment (pH<3). The impact of the medium pH on the antiproliferative potency of OMP has been recently discussed [13]. Here we used the “physiological” pH of the medium (7.4), because an acidic medium may influence the metabolic activity of the tumor cell. The fact that OMP is also effective when a neutral medium is used may be explained in two ways: OMP activation within or on intracellular acid compartments and/or interaction with a molecular target by the prodrug OMP. An acidic microenvironment of a solid tumour or culture medium acidification through lactate accumulation may, at least in part, enhance the activation of OMP and its pharmacodynamic properties.

The IC_50_ies of those human pancreatic cancer cell lines described here differ by a factor of approximately four. We assume that the pharmacodynamic differences shown in Figure 1 reflect differences in their transmembrane transport and metabolisation capacities.

OMP has been shown in the past to enhance the antitumoral effects of cytostatic drugs [10], [11]. The pharmacodynamic interaction assays confirmed that the cytostatic effects of 5-FU and GEM were also enhanced by OMP in a dose-dependent manner in the MiaPaCa-2 cell line. The cell lines ASPC-1, Panc-1 and PancTu-1, in contrast, showed a hormetic growth stimulatory effect of 5-FU, when it is given in doses below the IC_50_. The hormesis-phenomenon may contribute to chemoresistance, particularly when the plasmatic concentration of the drug declines exponentially after administration or when the tumorous microenvironment prevents sufficient tissue penetration [22], [23]. Nevertheless the molecular basis of this overcompensatory cell response enabling survival is not yet known. OMP reversed the hormesis-phenomenon in these three cell lines (Figure 2) and, therefore, may contribute to chemoresistance overcome in vivo. Panc-89 cells exhibited a seemingly paradoxical attenuation of the 5-FU mediated cytotoxicity by OMP, which would be classified as antagonism in the classical understanding of drug-drug-interaction. Nevertheless in this case there is no hormesis of 5-FU but of OMP itself, which is reflected in the dose-effect-curves. GEM did not reveal any hormetic effects, and the combination with OMP led to sligthly additive interactions (data not shown).

We chose the MiaPaCa-2 and ASPC-1 cell lines for further studies because of their different pharmacodynamics. The first step was to verify, whether lysosomal pH was elevated after OMP treatment [9], [11]. AO staining revealed that the lysosomal acidity, when related to the cytosolic pH, was even increased after treatment with either OMP or 5-FU+OMP in ASPC-1 cells and by 5-FU alone in MiaPaCa-2 cells. Only after 24 hours, we observed a slight, but significant suppression of the lysosomal/cytosolic acidity ratio in MiaPaCa-2 cells caused by OMP, 5-FU or a combination of both. The accumulation of acidic lysosomes within the cell as well as the elevation of the intralysosomal pH have both been recently described by Luciani et al. [10]. In addition we found that lysosomal acidification or alkealisation is not specific to OMP. Moreover OMP is known to be a far less potent vATPase inhibitor than bafilomycin, a specific vATPase inhibitor. OMP interacts mainly with cytoplasmic rather than luminal SH-groups, and then only at concentrations above 100 µM [47]. Accordingly, only a gradual increase in the lysosomal pH may be observed, if OMP accumulates within the cell. The fact, that 5-FU has the same effects, rather points to a regulatory cell function, i.e. stabilisation of cytosolic pH or decreased lysosomal transport and digestive capacities.

Electron microscopy was performed to further examine the subcellular morphologic changes. Treatment of ASPC-1 and MiaPaCa-2 cells with 80 µg/ml led to accumulation of phagophores and autophagosomes (Figure 4). When the dose was increased up to 160 µg/ml, apoptosis developed invariably in ASPC-1 cells, whereas in MiaPaCa-2 cells, early signs of apoptosis were already present after treatment with 80 µg/ml. Autophagy is a constitutive process of normal and transformed cells; but little is known about the initiation. Various reports suggest that autophagy may be induced by amino acid starvation [48] or energy depletion [41]. Autophagy has been associated with PCD following chemotherapy in apoptosis-resistant cells [49], [50]. In contrast, autophagy has also been described as a survival mechanism for malignant cells [51], [52]. Thus the therapeutic modulation of autophagy is considered to be a “double-edged sword” [53]. We hypothesise that the accumulation of the early markers of autophagy (phagophores and autophagosomes) after treatment with OMP reflects not only autophagy induction but also a disturbance of the lysosomal transport system and interferes with the survival function of autophagy, causing consecutively PCD.

Since our studies have so far shown that OMP treatment affects the lysosomal transport system and induces autophagy, we decided to analyse the metabolomics of both cell lines by proton NMR spectroscopy. OMP itself could be identified, but none of its known metabolites (i.e. hydroxyomeprazole and omeprazole acid) [54]. The different transmembrane transport capacities of ASPC-1 and MiaPaCa-2 cells rather than the metabolisation capacities seem to be responsible for the different pharmacodynamic characteristics of both cell lines.

The elevation of PUFA and decrease in the PC/Cho ratio after OMP treatment suggest induction of PCD and may also indicate catabolism of membranes and proliferation inhibition following down-regulation of choline kinase [36], [55] and upregulation of phospholipase A2 [34]. Since whole cell NMR spectroscopy detected a similar treatment response to 5-FU, these data are not specific to OMP.

To determine where OMP accumulated and how it caused PCD, we fractionated MiaPaCa-2 cells by ultracentrifugation and isolated the lysosomes. Proton NMR did not show any accumulation of OMP within this cell fraction, but Western Blot analysis which was performed for cell organelle identification unexpectedly showed that the lysosomes of the control were found in the first two fractions of the control group but only in the first (and lightest) fraction of the OMP group. Furthermore β-COP, the Golgi complex marker, diminished upon OMP treatment. Aniento et al. [56] reported that ß-COP which aggregates to transport vesicles of the Golgi complex in a pH dependent manner may be suppressed by bafilomycin A1 and nigericin thereby impairing the fusion process of early autophagosomes with lysosomes [57], [58]. Our results suggest a similar mechanism in MiaPaCa-2 cells, although the lysosomal pH was only slightly changed. However there is evidence that the vATPase functions not only as a proton pump but also as a pH-sensor [59]. Bafilomycin and Concanamycin, which interact with the V-subunit of the vATPase, prevent the maturing and fusion of lysosomes. This Vsubunit is therefore believed to take part in the protein sorting function of lysosomes [60]. We hypothesise that a reversible interaction of OMP with the vATPase can affect the fusion of the lysosomes with autophagosomes even without its complete inhibition. This would explain the results of the Western blot analysis and why OMP is not detectable by NMR spectroscopy within lysosomes but in the whole cell spectra.

Furthermore LC3-Western blot analysis was performed in order to substantiate the specificity of autophagy for OMP and to discriminate the quantitative changes of autophagy induction from those of autophagosome turnover. We found that the LC3-II signal, which indicates the autophagy turnover, was elevated in a dose dependent manner by OMP, but not 5-FU (Figure 9). The LC3-I fraction was also induced but the ratio of both was not quantified because of the known artifacts of LC3-I [37]. We conclude that OMP induces autophagy and also modulates the turnover causing accumulation of autophagosomes as described above. Interestingly, morphologic signs of autophagy and apoptosis coexist at higher doses (80 and 160 µg/ml) of OMP raising the question how both processes are interacting. It is now commonly believed that autophagy itself does not cause apoptosis but rather serves as an accompanying or frustran survival process [38]. If this would be the case, OMP should have influenced apoptosis-controlling genes directly or indirectly.

MRNA quantification revealed the downstream effects including upregulation of the proapoptotic bad gene and down-regulation of the anti-apoptotic bcl-2 and bcl-xL genes 24 hours after treatment of ASPC-1 cells with 5-FU and/or 5-FU+OMP (Figure 10). The mdr-1 gene was up-regulated after OMP treatment indicating another aspect of the complexity of OMP-mediated effects. The pro-survival gene survivin was down-regulated by OMP.An association of Pgp inhibition and survivin down-regulation was also reported by Liu et al. [61]. In contrast the mdr-1 gene was not upregulated in MiaPaCa-2 cells. The vATPase was up-regulated 24 hours after beginning of treatment with 5-FU+OMP. The Western blot analyses shown in Figure 12 confirm these observations.

We conclude that OMP inhibited cancer cell proliferation by interfering with the intrinsic autophagy of ASPC-1 and MiaPaCa-2 cells via transmembrane transport activation (ASPC-1) as well as a disrupted lysosomal transport (MiaPaCa-2). Complex biochemical effects caused by OMP as well as 5-FU can be summarised as changes in phospholipid and membrane metabolism that suggest the start of PCD. The differences between the cell lines are gradual and reflect the higher effectivity of OMP in MiaPaCa-2 cells. Unlike 5-FU, OMP caused no direct involvement of those apoptosis-controlling pathways investigated here. The coexisting signs of apoptosis and autophagy activation suggest however that the autophagy modulation caused by OMP may well be the starting mechanism or even a part of the PCD itself.

As neither the process of autophagy itself nor its role in overcoming chemotherapy resistance is understood, further studies, directed at the immediate autophagy induction pathway, are required.

The clinical relevance of our findings is supported by the fact that autophagy modulation may circumvent the antiapoptotic resistance mechanisms characteristic of many tumor entities. Since OMP has already been established in clinical practice these results could rapidly lead to the development of new therapeutic strategies in the treatment of pancreatic cancer.

## Materials and Methods

### Cell lines

The human pancreatic cancer cell lines MiaPaCa-2 [21], ASPC-1 [19], Panc1 [62], Colo357 [63], PancTu1 [64], Panc89 (identical to T3M-4) [65] were cultured with RPMI/ DMEM (1∶1, Invitrogen, Karlsruhe, Germany) and were supplemented with 2 mM glutamine, Penicillin/Streptomycin 1% (Invitrogen, Karlsruhe, Germany), and 10% FCS (PAA, Cölbe, Germany) at 37°C, 5% CO2.

### Drugs

5-FU and OMP (Astra Zeneca, Wedel, Germany) were obtained from the University's hospital pharmacy. 5-FU was dissolved in 0.9% NaCl and stored in the dark at 20°C. OMP was stored as dry substance at room temperature and dissolved in the medium directly before use. Medium and drugs were exchanged daily.

### Antibodies

The following primary antibodies were used: Cathepsin D, Lamp-1 (both obtained from Cell Signalling Inc., Danvers, USA), β-COP (abcam, Cambridge, UK), Bad (R&D Systems, Minneapolis, USA), Puma, Atg12 (both obtained from biomol, Hamburg, Germany), LC3 (mAB2G6 Enzo Life Sciences, USA), and actin (mAB, Sigma, USA).

### ATP-bioluminescence assays

Determination of the IC_50_ies: Cells were seeded in 96 well plates, 1000 viable cells/well, and cultured as described above. In order to determine the IC_50_ of 5-FU and OMP, we used 1 control well and 23 wells with inceasing concentrations of each drug, which were added 24 h after cell seeding. The ATP content in each well was determined 4 days later using the HS II ATP-Bioluminescence Kit (La Roche, Germany). Bioluminescence was measured with the Lumistar luminometer (BMG Labtech GmbH, Offenburg, Germany). The IC of each drug was calculated using the three parameter log-logistic model of the R package “drc” [66], [67]. The biphasic (hormetic) dose-effect-curves of the drug combinations of 5-FU and OMP in various cell lines could not be fitted using this modell. In these cell lines the Brain-Cousens-modell with 3 parameters was applied [28].

### Acridine Orange (AO) Fluorescence Microscopy

10^4^ MiaPaCa-2 and ASPC-1 cells were seeded in 5 cm^2^ plates and incubated for 24 hours. OMP 80 µg/ml , 5-FU 5 µg/ml or both were then added, and after 30 min or 24 hours of further incubation, AO (5 µg/ml) was added to the living cells for 15 min. The medium was then exchanged. Fluorescence analyses were performed at 525 nm (green) and 650 nm (red) on an Olympus microscope. Three non-overlapping digital pictures per plate with a resolution of 1376×1038 pixels were taken and 3 plates per group (control, OMP, 5-FU or combination) were grown up. The brightness value was calibrated to 1–256 for each channel (red, green, blue).

The CellProfiler software (Broad Institute, Cambridge, UK) was used for the image processing [68]. Cells and lysosomes were identified using the “ExampleSpeckleImages” pipeline with some minor adaptations for both cell lines. The red fluorescence indicating acid compartments (mainly lysosomes) and the green fluorescence showing the physiologic pH of the cytosol were integrated for each cell, and the red-green-ratio was determined. The integral ratios per cell of the 3 treatment regimens were compared to control for each time and cell line by the Students t-test.

### Transmission Electron Microscopy (TEM)

2×10^3^ adherent MiaPaCa-2 or ASPC-1 cells per well, respectively were seeded on 6 well plates and incubated for 24 hours. After adding 80 µg/ml OMP, 5 µg/ml 5-FU or the combination of both, cells were incubated for additional 24 hours before fixation with 2.5% glutaraldehyde (Fluka, Germany) containing 1.5% saccharose in PBS and post-fixation in 2% aqueous osmium tetroxide (Fluka, Germany). The samples were then dehydrated in a 1-propanol series, block stained in 1% uranyl acetate, and embedded in Epon (Fluka, Germany). Ultra thin sections of 80 nm were contrasted with 0.3% lead citrate and imaged in a Philips EM400 TEM. Three wells per cell line and treatment group were used (overall 24). Ten cells per well were selected randomly and lysosomes and late autophagosomes were counted (30 cells per group). Mean numbers were compared by the Students T-test after confirming normal distribution by the F-Test.

### Nuclear magnetic resonance (NMR) spectroscopy

MiaPaCa-2 and ASPC-1 cells were cultured in 20 cm culture plates using DMEM without Hepes as medium. Drugs were added two days after seeding (80 µg/ml OMP, 5 µg/ml 5-FU or both). Measurements were performed 24 h after adding the drugs. After washing the cells twice with PBS solution they were harvested from the plates, centrifuged with 1000 U/min for 5 min, resuspended in 0.4 ml D2O and transferred into the tubes.Three 5-mm-NMR-tubes were prepared for each group, each of them containing the cells of three 20-cm- culture-plates. The cells were kept at 20°C during preparation and measurement. It was verified that neither qualitative nor quantitative changes occurred in the spectra, when the cells were stored at this temperature. Spectroscopy was performed on a 600 MHz Bruker spectrometer with 128 scans, 3.3 s repetition time and water suppression. The spectra were analyzed and the signals integrated with Win-NMR 1D software (Bruker, Karlsruhe, Germany). The Fourier transformation was performed without LP filling and apodization. Signal identification was based on peak spiking and literature. We used the methyl doublet of lactate as a chemical shift reference at 1.3 ppm.

The statistical calculation and the network creation was done using the R package “Rgraphviz” [67], [69].

### Lysosome enrichment by ultracentrifugation of MiaPaCa-2 cells

MiaPaCa-2 cells were cultured in DMEM without Hepes. After two days OMP 80 µg/ml was added to the medium for additional 24 hours in the treated group. Cells were lysed by douncing. The Lysosome enrichment kit (Pierce, Rockford, USA) was used for lysosomal purification following the instructions of the manufactor as described elsewhere before [70]. About 10 cells were used for each of the two groups: control and OMP 80 µg/ml six hours after beginning of treatment. Ultracentrifugation was performed for 30 min at 18000 G. The ultracentrifugates were washed twice before NMR spectroscopy according to the instructions of the manufacturer. NMR spectroscopy was performed as described in subsection “Nuclear magnetic resonance (NMR) spectroscopy”.

### Western blot analysis

MiaPaCa-2 and ASPC-1 cells were incubated 24 hours either untreated or treated with bafilomycin A1 (1 µM) for two hours, or with various concentrations of OMP (40, 80, 160 µg/ml), 5-FU (5 µg/ml) or the combination of OMP 80 µg/ml and 5-FU 5 µg/ml for 24 hours.

Cells extracts were clarified by centrifugation at 15000 g for 20 min at 4°C and the protein concentration of the lysates was determined using the BCA (bicinchoninic acid) protein assay (Pierce, Rockford, USA). In each case, equal protein amounts, or equal volumes of single fractions derived from lysosome enrichment of control and OMP treated cells (see subsection 4.8) were loaded on 12.5% SDS/-gels and transferred onto a Hybond C super nitrocellulose blotting membrane (GE Healthcare). The membranes were blocked in TBS containing 0.1% (v/v) Tween 20 and 5% (w/v) non-fat milk for 1 h before being probed with primary rabbit polyclonal antibodies either specific for Bad (1∶1000 dilution, R&D, USA, 1∶600), PUMA (Epitomics, CA, USA, 1∶1000 dilution), Atg12 (Biomol, Hamburg, Germany, 1∶200 dilution), LAMP-1 (1∶500 dilution, β-COP (1∶1000), Cathepsin D (1∶1000 dilution), LC3 (0.5 µg/ml) or actin (Sigma, 1∶10000 dilution) . Immuncomplexes were detected by incubation for 45 min with the appropriate HRP-conjugated secondary antibody (1∶1000 dilution), followed by ECL (enhanced chemiluminescence) detection (GE Healthcare). Each of these investigation was repeated three times.

### mRNA quantification

The cells were seeded on 10 cm^2^ culture plates and incubated as described above for 24 hours. Drugs were added as described above. Three plates per cell line, time point and treatment group were used. After 0 (control), 6, 12, 18 and 24 hours, the cells were trypsinized, kept on ice and washed twice with PBS. Thereafter, they were kept at −80°C till mRNA extraction. Bad, bax, bcl-2, bcl-xl, v-ATPase, mdr-1, mrp, survivin and gapdh transcripts were measured from cDNA in duplicate experiments using ready-to-use PCR testkits (Roboscreen Gesellschaft für molekulare Biotechnologie, Leipzig, Germany) [62].
